# Self-Renewal of Acute Lymphocytic Leukemia Cells Is Limited by the Hedgehog Pathway Inhibitors Cyclopamine and IPI-926

**DOI:** 10.1371/journal.pone.0015262

**Published:** 2010-12-28

**Authors:** Tara L. Lin, Qiuju H. Wang, Patrick Brown, Craig Peacock, Akil A. Merchant, Sarah Brennan, Evan Jones, Karen McGovern, D. Neil Watkins, Kathleen M. Sakamoto, William Matsui

**Affiliations:** 1 Section of Hematology and Oncology, Department of Internal Medicine, LSU Health Sciences Center, New Orleans, Louisiana, United States of America; 2 Sidney Kimmel Comprehensive Cancer Center, Johns Hopkins University School of Medicine, Baltimore, Maryland, United States of America; 3 Infinity Pharmaceuticals, Cambridge, Massachusetts, United States of America; 4 Gwynne Hazen Cherry Memorial Laboratories, Department of Pediatrics, Jonsson Comprehensive Cancer Center, David Geffen School of Medicine at University of California Los Angeles, Los Angeles, California, United States of America; Yale Medical School, United States of America

## Abstract

Conserved embryonic signaling pathways such as Hedgehog (Hh), Wingless and Notch have been implicated in the pathogenesis of several malignancies. Recent data suggests that Hh signaling plays a role in normal B-cell development, and we hypothesized that Hh signaling may be important in precursor B-cell acute lymphocytic leukemia (B-ALL). We found that the expression of Hh pathway components was common in human B-ALL cell lines and clinical samples. Moreover, pathway activity could be modulated by Hh ligand or several pathway inhibitors including cyclopamine and the novel SMOOTHENED (SMO) inhibitor IPI-926. The inhibition of pathway activity primarily impacted highly clonogenic B-ALL cells expressing aldehyde dehydrogenase (ALDH) by limiting their self-renewal potential both *in vitro* and *in vivo*. These data demonstrate that Hh pathway activation is common in B-ALL and represents a novel therapeutic target regulating self-renewal and persistence of the malignant clone.

## Introduction

During embryonic development, conserved signaling pathways such as Hedgehog (Hh), Wingless (Wnt) and Notch precisely regulate morphogenesis by dictating cell fate decisions such as self-renewal and differentiation [1]. These pathways are subsequently silenced in most adult tissues but frequently reactivated in a wide range of human malignancies. It has been hypothesized that malignant transformation recapitulates many developmental processes, and signaling pathways required for the normal development of a specific tissue or organ may be aberrantly activated in the corresponding malignancy. For example, the Notch pathway is required for normal T cell development, and deregulated Notch signaling is a common feature of T-cell acute lymphocytic leukemia (ALL) [2]. Preliminary reports also suggest that Hedgehog signaling promotes the growth of T-ALL [3], [4]. The role of developmental signaling pathways in the pathogenesis and self-renewal of B cell ALL (B-ALL) is not clear, but recent data suggest that the Hh signaling pathway is involved in the development of B cell precursors from primitive hematopoietic stem cells, as well as self-renewal and cell survival in human B cell malignancies [5]–[8].

In mammalian cells, key components of the Hh signaling pathway have been identified. The three Hh ligands (Sonic, Indian and Desert) activate pathway signaling by binding to the cell surface receptor Patched (PTCH). In the unbound state, PTCH exerts an inhibitory effect on Smoothened (SMO). However, when PTCH is bound to ligand, this inhibition is released and eventually modulates the activities of the three GLI transcription factors (GLI1-3) at target promoters. Evidence that the Hh pathway is involved in human cancers is exemplified by Gorlin syndrome arising from autosomal dominant mutations in *PTCH1* that dramatically increase the risk of advanced basal cell carcinoma (BCC), medulloblastoma and rhabdomyosarcoma [9]. Spontaneous mutations in *PTCH* and *SMO* that confer aberrant pathway activity are also commonly found in spontaneous cases of BCC and medulloblastoma. Increased Hh signaling has been described in a wide range of other human cancers, including chronic myeloid leukemia (CML), multiple myeloma (MM), pancreatic cancer, glioblastoma, prostate cancer, breast cancer and small cell lung cancer [6], [8], [10]–[14]. The vast majority of these tumors lack mutational activation of Hh pathway components, and increased Hh signaling may be due to over-expression of activating ligands or SMO [15], [16]. In pre-clinical models, pathway inhibition may result in reduced tumor cell proliferation or survival. Evidence that the Hh signaling pathway plays a role in several B cell malignancies including MM and non-Hodgkin lymphoma (NHL) as well as normal early B-cell development suggests that it may be involved in precursor B-ALL [6], [8], [17]. Moreover, in several human hematologic malignancies, the Hh signaling pathway has been found to regulate self-renewal required for long-term maintenance of the malignant clone [6], [18],[19]. We examined Hh signaling pathway activity in B-ALL and found that Hh signaling regulates the self-renewal of highly clonogenic tumor cells both *in vitro* and *in vivo*. These results suggest that the inhibition of Hh signaling may improve long-term outcomes in B-ALL by targeting the clonogenic cells responsible for disease relapse.

## Materials and Methods

### Ethics Statement

Primary clinical specimens were obtained from patients with newly diagnosed or relapsed B-ALL. Normal bone marrow CD34^+^ CD19^+^ progenitors from normal bone marrow donors were used as controls in real-time PCR experiments. All patients granted written informed consent as approved by the UCLA Institutional Review Board (Medical Institutional Review Board 2).

### Cells and cell culture

Human precursor B-ALL cell lines were obtained from the American Type Tissue Collection (REH, RS4;11) and the DMSZ German Collection of Microorganisms and Cell Culture (Nalm 6, SEM-K2, TOM-1). The HB-1119 cell line was a gift of M. Cleary (Stanford University). Cells were maintained in Advanced RPMI (Invitrogen) containing 1% fetal bovine serum (FBS) and L-glutamine. Primary clinical specimens were obtained from patients with newly diagnosed or relapsed B-ALL. Normal bone marrow CD34^+^ CD19^+^ progenitors from normal bone marrow donors were used as controls in real-time PCR experiments. All patients granted informed consent as approved by the UCLA Institutional Review Board. Mononuclear cells were isolated from freshly harvested bone marrow aspirates or peripheral blood by density centrifugation (density <1.078, Ficoll Paque, Pharmacia) followed by two washes with RPMI.

### Expression of Hh pathway components

The expression of Hh pathway components *DHH, IHH, SHH, HHIP, GAS, PTCH1, PTCH2, SMO, GLI1, GLI2, GLI3* in precursor B-ALL cell lines was detected by reverse-transcriptase PCR. Human fetal brain (HFB) was used as a positive control for Hh pathway expression, and â-actin was used as a control gene for experiments with cell lines and HFB with and without reverse transcriptase. Levels of *PTCH1*, *SMO* and *GLI1* were measured in cell lines and primary clinical specimens by real-time quantitative PCR using the Step 1 Plus thermal cycler and Fast Taqman reagent (Applied Biosystems). Clinical specimens which expressed all three genes *PTCH1*, *SMO* and *GLI1* were considered to positive for expression of Hh pathway components. Normal bone marrow CD34^+^ CD19^+^ progenitors from normal bone marrow donors were used as controls in real-time PCR experiments. Quantitative calculations were performed using the ÄÄ_ct_ method. Primer sequences are listed in Supplemental Table S1.

### Hh pathway agonists and inhibitors

Recombinant Sonic Hedgehog (ShhNP) was a gift of P. Beachy (Stanford University). The monoclonal antibody 5E1 was obtained from the Iowa Hybridoma Bank [20]. The naturally occurring SMO inhibitor cyclopamine and the semi-synthetic cyclopamine derivative IPI-926 were provided by Infinity Pharmaceuticals [21].

### Transient transfection studies

REH and RS4;11 precursor B ALL cells were co-transfected with a Gli-responsive firefly luciferase vector containing 8 tandem copies of a consensus Gli binding site immediately upstream of the chicken lens crystallin promoter (pGL3-8×-Gli-luciferase) and constitutive *Renilla* luciferase expression vectors (pRL-CMV; Promega) using the Amaxa Nucleofector Apparatus (Lonza) [22]. Transfected cells were then treated with Hh pathway modulators for 48 hours. Treated cells were then harvested and assayed for firefly and *Renilla* luciferase activities using the dual luciferase reporter assay (Promega).

### Clonogenic assays

REH and RS4;11 cells were seeded at 1×10^5^ cells/ml and treated with ShhNP, 5E1, cyclopamine (5 µM) or IPI-926 (1 µM) for 72 hours. Following 72 hours of treatment, cells were washed twice with media to remove drugs then 500 cells were plated in quadruplicate in 1 ml of 1.2% methylcellulose, 30% FBS, 1% bovine serum albumin (BSA), 0.1 mM 2-mercaptoethanol, and 2 mM L-glutamine. Samples were plated in quadruplicate onto 35 mm^2^ tissue culture dishes and incubated in a humidified atmosphere at 37°C and 5% CO_2_. Colonies consisting of >40 cells were counted using an inverted microscope at 10–14 days, then harvested and replated in methylcellulose.^20^ Results represent colony formation during each round of replating relative to vehicle control cells.

### Examination of ALDH activity by flow cytometry

REH and RS4;11 cells were evaluated for aldehyde dehydrogenase (ALDH) activity using the Aldefluor reagent (Stem Cell Technologies) according to the manufacturer's instructions followed by flow cytometry with a FACSCalibur flow cytometer. Viable cells were distinguished by the lack of staining with propidium iodide (1 µg/ml) and considered positive for ALDH based on a control staining reaction using the enzyme inhibitor diethylaminobenzaldehyde (DEAB). Cell sorting was performed using a FACSAria flow cytometer, and viable cells with the lowest 5% Aldefluor staining were considered ALDH negative.

### NOD/SCID mice

All animal experiments were conducted in accordance with protocols approved by the Johns Hopkins Institutional Animal Care and Use Committee. For *in vivo* treatment studies, NOD/Scid mice were injected with 10^4^ REH cells by tail vein 18–24 hours after irradiation (300 cGy). The day after cell injection, mice were treated twice a week for 3 weeks with the SMO inhibitor IPI-926 (40 mg/kg IP), then followed until the development of symptoms (weight loss or paralysis) and sacrificed for analysis. Harvested bone marrow and spleen cells were stained with antibodies directed against human CD19 and CD45 (BD Biosciences) followed by flow cytometry. Animals were considered positive for engraftment if human CD45^+^CD19^+^ cells were >1% of cells. For secondary transplants, bone marrow cells (10^5^ total human CD19^+^ cells) were injected by tail vein into irradiated NOD/Scid mice as above. No treatment was administered to secondary recipients. For *in vitro* treatment studies, REH cells were initially treated in culture with IPI-926 (1 µM) for 14 days. Cells were harvested, washed twice with media then injected by tail vein into irradiated NOD/Scid mice. Mice were observed until the detection of symptoms and analyzed as above.

### Statistical analysis

Data are expressed as the mean ± standard error of the mean (SEM). Comparisons between treatments were performed using a two-tailed, paired Student's *t* test. Mouse studies were analyzed using a Kaplan-Meier analysis and the log-rank test. For all analyses, P<0.05 was considered statistically significant.

## Results

### Human precursor B-ALL cell lines and clinical samples express Hedgehog pathway components

**Figure 1:**
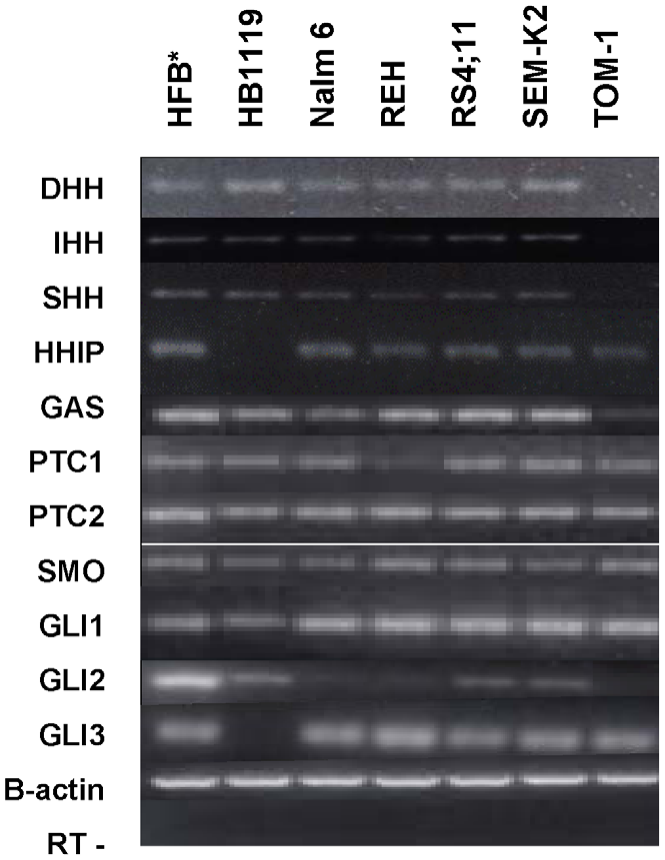
Expression of Hh pathway components is common in precursor B-ALL cell lines. Qualitative RT-PCR analysis of Hh pathway gene expression in precursor B-ALL cell lines. *HFB*, human fetal brain, used as positive control for all experiments. For each cell line, positive and negative control reactions were run with B-actin primers with (to demonstrate equal amounts of cDNA template across each cell line) and without (to rule out contamination with genomic DNA) reverse transcriptase. B-ACTIN- denotes PCR results from mock cDNA synthesis reactions lacking RT. Precursor B-ALL cell lines: HB 1119 (t11;19), Nalm6 (t2;6), REH (t12;21), RS4;11 (*MLL* gene rearrangement); SEM-K2 (*MLL* gene rearrangement), TOM-1 (Ph^+^). Electrophoresis was performed with each gene separately across the panel of cell lines. The separate gels are shown together for the purpose of clarity across cell lines in the figure.

B-ALL is a heterogeneous disorder with several cytogenetic variants that correlate with distinct clinical outcomes. We examined a panel of human precursor B-ALL cell lines reflecting this heterogeneity for expression of Hh pathway components by RT-PCR and found that each expressed several of the Hh pathway components (Figure 1). All cell lines in our panel expressed *GAS, PTCH1, PTCH2, SMO* and *GLI1* and a majority of cell lines expressed Hh ligands, *HHIP, GLI2* and *GLI3.* Moreover, expression of the Hh target genes *PTCH1* and *GLI1* suggest that Hh signaling was active in these lines. We extended these studies and quantified the expression of *PTCH1, SMO* and *GLI1* by each of these cell lines as well as primary B-ALL clinical specimens using real-time PCR (Table 1). Compared to previous studies in MM in which only a fraction of primary tumors expressed Hh pathway components [6], [23] a high proportion of the primary B-ALL specimens (39/41, 95%) expressed all three genes *PTCH1, SMO* and *GLI1* (noted as “Hh pathway expression” in Table 1). Moreover, expression was detected in specimens that encompassed the most curable (TEL-AML1), most common (normal cytogenetics) and poorest prognosis (Ph^+^) ALL subtypes (Table 1), although the quantitative expression of Hh pathway components did not correlate with any specific B-ALL subtype (data not shown).

**Table 1:** Hh pathway components are commonly expressed in primary clinical specimens across cytogenetic and prognostic subgroups of precursor B-ALL.

ALL Cytogenetic Subtype	Hh pathway expression
TEL-AML1 translocation	9/10
Normal karyotype	17/17
Hyperdiploid karyotype	5/5
Philadelphia chromosome	2/2
Other cytogenetic findings	6/7
**TOTAL**	**39/41**

Expression of Hh pathway components *PTCH1, SMO* and *GLI-1* was measured by quantitative RT-PCR in a panel of 41 clinical specimens from patients with newly diagnosed or relapsed ALL from various cytogenetic subtypes. *Hh pathway expression* was defined as those specimens with expression of all three genes (*PTCH1, SMO* and *GLI1*) and was seen in the majority of clinical specimens (39/41). Expression of Hh pathway components was seen across all cytogenetic subtypes, including those with good and poor prognosis.

### Hedgehog signaling is active in precursor B-ALL and inhibited by cyclopamine and IPI-926

**Figure 2:**
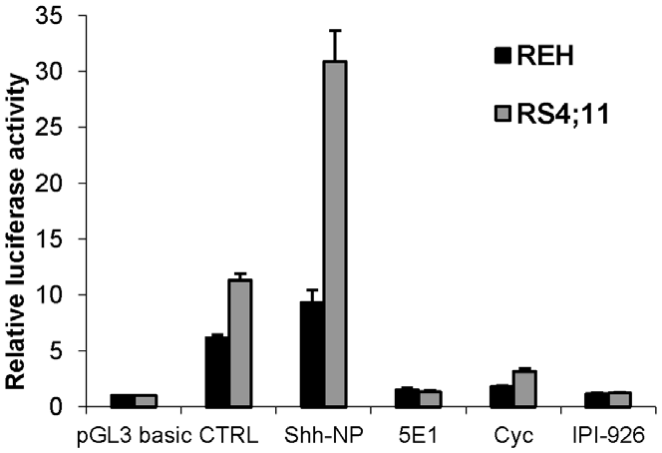
Hh pathway is active and inhibited by the SMO-inhibitors cyclopamine and IPI-926. Precursor B-ALL cell lines REH and RS4;11 were transiently transfected with a Gli-responsive luciferase reporter or pGL3-basic, a plasmid lacking GLI binding site, as a negative control. Relative luciferase levels were measured following 48 hours of treatment with vehicle control, purified, dual lipid-modified Shh ligand (Shh-NP), SHh antibody 5E1 (10 µg/ml), cyclopamine (5 µM) or IPI-926 (1 µM). Hh pathway stimulation with SHh (p<0.005) and inhibition with 5E1 (p<0.0001), cyclopamine (p<0.0005) and IPI-926 (p<0.0002) was statistically significant in both the REH and RS4;11 cell lines.

In order to determine whether the Hh signaling pathway is active in B-ALL, we carried out transient transfection experiments with a GLI-responsive luciferase reporter and studied REH and RS4;11 cells since they are representative of good and poor risk ALL, respectively. Both cell lines demonstrated significantly higher baseline reporter activity compared to a control plasmid that lacked GLI binding sites (Figure 2, p<0.0004). Next, we examined the effects of HH pathway modulation at the level of the Hh ligand by stimulation with the recombinant Hh ligand SHh-NP and Hh pathway inhibition by the monoclonal antibody against the Hh ligand, 5E1. Cells were treated for 48 hours with SHh-NP or 5E1. Reporter activity was enhanced by exogenous SHh ligand (p<0.005) and inhibited by the ligand-neutralizing monoclonal antibody 5E1 (p<0.0001), suggesting that Hh signaling was ligand dependent and not likely due to activating mutations within pathway components. We also studied the inhibitory activity of two SMO inhibitors, cyclopamine and IPI-926 [21]. Following 48 hours of treatment, both these inhibitors significantly inhibited luciferase reporter activity (cyclopamine, p<0.0005, IPI-926, p<0.0002), suggesting that the activity of the Hh signaling pathway could be externally modulated in B-ALL.

### Hh pathway inhibition limits clonogenic growth of precursor B-ALL cell lines

**Figure 3:**
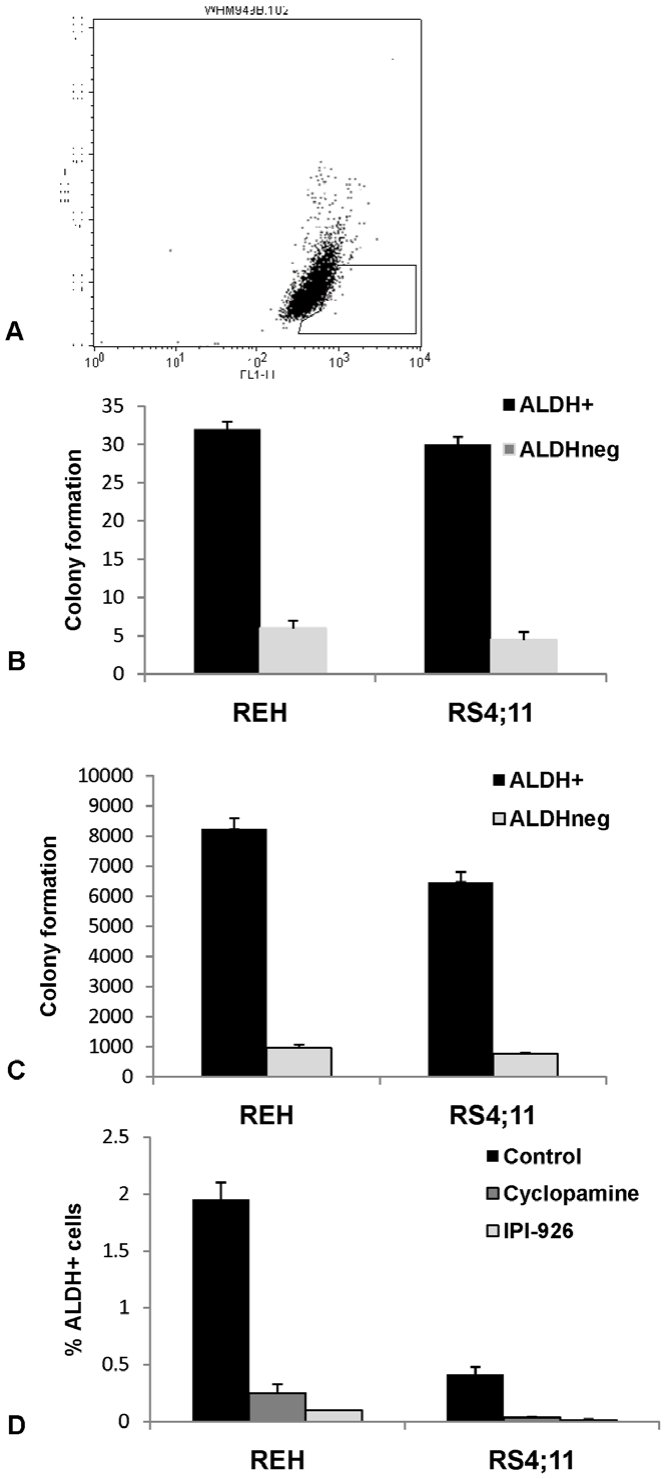
ALDH^+^ cells are enriched for self-renewal potential and targeted by IPI-926. (A)ALDH^+^ and ALDH^neg^ cells were isolated from the REH cell line by FACS using the Aldefluor reagent and DEAB control. The gated area represents the ALDH^+^ cells, measuring 1.18% of total REH population. (B) ALDH^+^ and ALDH^neg^ cells from REH and RS4;11 were plated in methylcellulose to evaluate clonogenic growth. Colony formation was measured at 10–14 days. There was a 5-fold difference in colony number following initial plating for REH (p = 0.05) and RS4;11 (p<0.01). (C) A representative plate from initial plating was washed with media and cells resuspended prior to replating in methylcellulose for an additional 10-14 days. Secondary plating of ALDH^+^ and ALDH^neg^ cells demonstrated an 8-fold difference in colony-formation between the ALDH^+^ and ALDH^neg^ cell fractions for REH (p<0.002) and RS4;11 (p<0.001). (D)Unfractionated REH and RS4;11 cells were treated with cyclopamine (5 µM), IPI-926 (1 µM) or vehicle control for 10 days and the percentage of ALDH^+^ and ALDH^neg^ cells was measured using the Aldefluor staining kit and FACS. The reduction in ALDH^neg^ cells following treatment with cyclopamine and IPI-926 was statistically significant for REH (p<0.01) and RS4;11 (p<0.03).

We previously demonstrated that aberrant Hh signaling plays a role in the self-renewal of MM and glioblastoma [6], [11], and similar findings have been reported in CML [18], [19]. In these studies, self-renewal was attributed to distinct populations of tumor cells termed tumor initiating cells or cancer stem cells (CSC). CSC have been reported in B-ALL, but a universal phenotype has not been described and is likely to vary according to the specific B-ALL subtype.^16–21^ Tumorigenic cells have been identified using specific surface antigen expression in most diseases, but stem cells may be isolated from normal tissues based on processes conferring drug resistance such as ABCG2 and aldehyde dehydrogenase (ALDH) [24]–[26]. Relative ALDH activity can identify highly clonogenic cell populations in a number of B cell malignancies, such as MM and Hodgkin lymphoma [27], [28], and we found that the REH and RS4;11 cell lines contained small populations (∼1%) of ALDH^+^ cells (Figure 3A). We isolated ALDH^+^ and ALDH^neg^ cells from each line by FACS and found that ALDH^+^ cells formed 5–7 fold more colonies than ALDH^neg^ cells in methylcellulose (Figure 3B, p = 0.05 for REH, p<0.01 for RS4;11). Moreover, this difference significantly increased during serial replating (Figure 3C, p<0.002 for REH, p<0.001for RS4;11), a surrogate for self-renewal potential. Therefore, ALDH expression may be associated with increased self-renewal potential in B-ALL.

**Figure 4:**
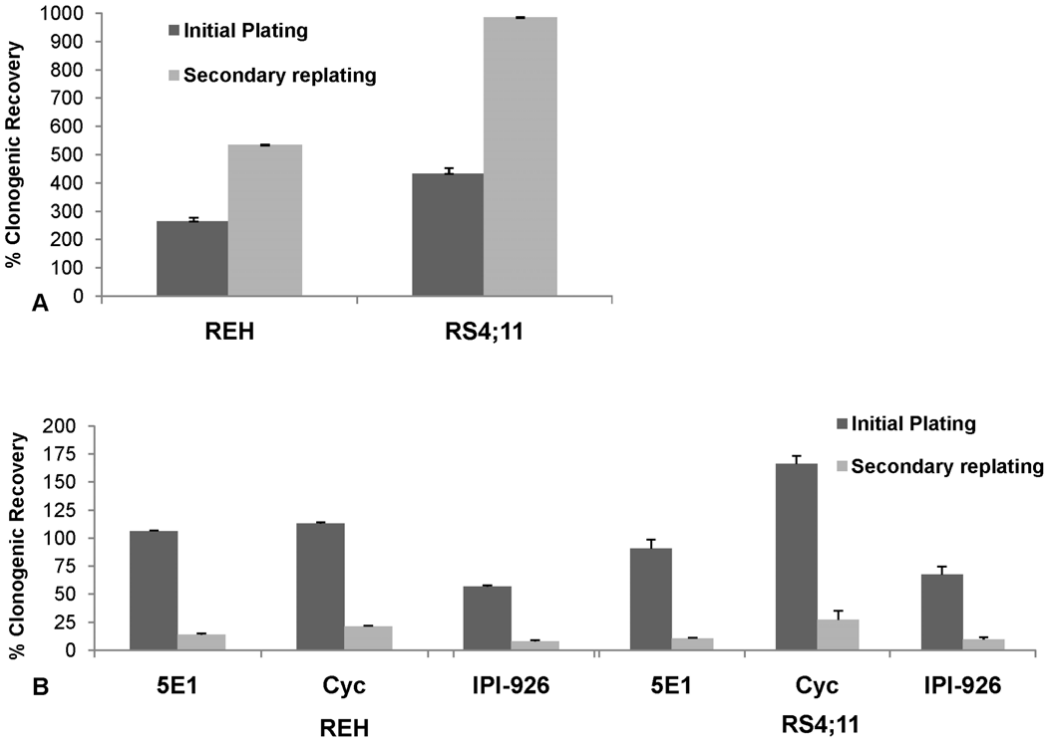
Hh pathway regulates self-renewal in precursor B-ALL. Clonogenic recovery of REH and RS4;11 cells following treatment with Hh pathway agonist and inhibitors. B-ALL cells were treated with Hh pathway modulators for 72 hours, washed free of drug and plated in quadruplicate in methylcellulose. At 10–14 days, colonies were counted (represented as initial plating). A representative plate was then washed and cells resuspended and replated. After an additional 10–14 days, colonies were counted (represented as secondary replating). Clonogenic recovery of untreated cells was normalized to 100% and plating results from all treatment groups are expressed as % control ± SEM. (A)Colony-formation of REH and RS4;11 cells following treatment with the Hh pathway ligand SHhNP. Increased colony-formation was statistically significant in both cell lines at initial and secondary replating despite no further addition of drug (p<0.001 for all groups). (B) Colony-formation of REH and RS4;11 cells following treatment with Hh pathway inhibitors 5E1 (10 µg/ml), cyclopamine (5 µM), IPI-926 (1 µM). Inhibition of clonogenic growth at secondary replating for both REH and RS4;11 cells was statistically significant (p<0.02) with Hh pathway inhibition with 5E1, cyclopamine and IPI-926.

In order to determine whether Hh pathway activity plays a role in regulating the self-renewal of precursor B-ALL cells, REH and RS4;11 cells were treated with cyclopamine or IPI-926 and then analyzed for ALDH activity. Both drugs significantly reduced the frequency of ALDH^+^ cells in each cell line compared to control vehicle treated cells, suggesting that the Hh pathway activity maintains this self-renewing population of cells (Figure 3D, p<0.01 for REH, p<0.03 for RS4;11). We also examined the effects of Hh pathway modulation on *in vitro* clonogenic growth and treated REH and RS4;11 cells with SHh-NP, 5E1, cyclopamine or IPI-926. Following 72 hours of treatment, SHh-NP significantly increased colony formation both during primary and secondary plating (Figure 4A, p<0.001). In contrast, Hh pathway inhibition by 5E1 or either SMO inhibitor had no or only minor effects on colony formation after initial plating compared to vehicle treated control cells. However, upon serial replating (without further exposure to drug), secondary colony formation was significantly inhibited by each of these agents (Figure 4B, p<0.02 for REH and p<0.001 for RS4;11). Moreover, the effects of Hh pathway inhibition were long-lasting as decreased colony formation was maintained for at least 28 days following initial treatment.

### Hh inhibition with IPI-926 limits B-ALL self-renewal *in vivo*

**Figure 5:**
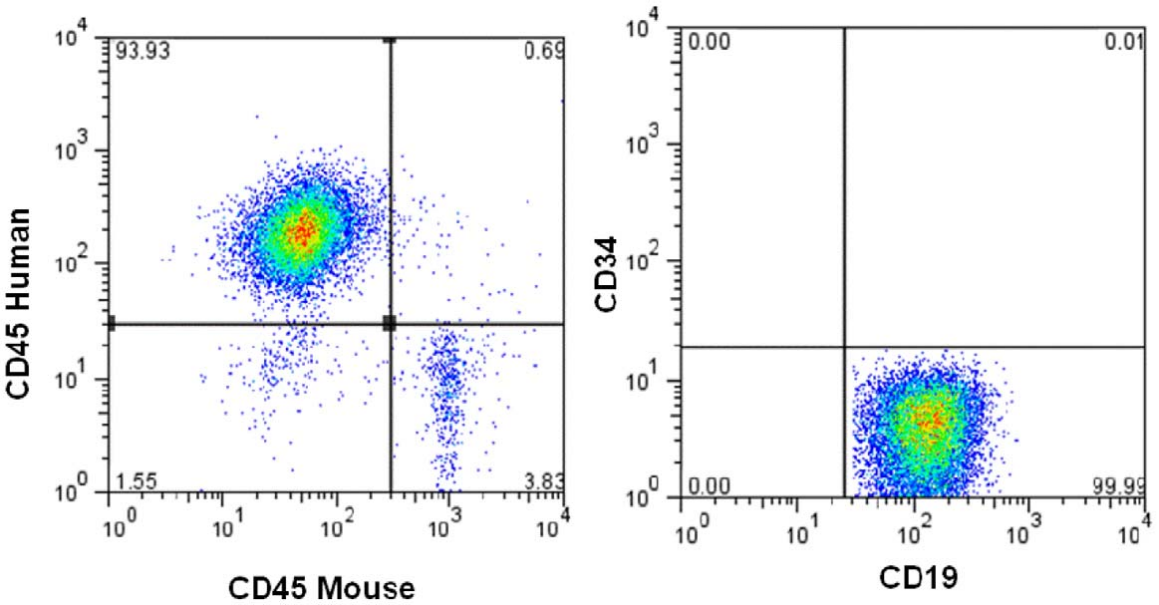
Engraftment of human leukemia cells in NOD/Scid mice. Representative flow cytometry results of mouse bone marrow demonstrating engraftment of human leukemia. Mice were injected with REH cells and then treated with either vehicle control or IPI-926 for 21 days. Mouse bone marrow was harvested after development of symptoms and analyzed by flow cytometry for the presence of human leukemia cells. All mice in both treatment groups had engraftment of human leukemia cells that were mouse CD45 negative, human CD45 positive and human CD19 positive.

Primary colony-formation assays in methylcellulose demonstrate effects on tumorigenic potential of these leukemia cells. Our finding that the effects of Hh inhibition on leukemic growth persisted through serial replating without further exposure to drug suggests that Hh signaling is involved in the regulation of long-term self-renewal as well. To further investigate the effects of Hh inhibition on self renewal and the relevance of the Hh signaling pathway as a therapeutic target in human B-ALL, we examined the activity of IPI-926 against REH cells *in vivo*. NOD/Scid mice were injected with REH cells then treated with IPI-926 or vehicle control for 21 days. There was no significant difference in median survival between the untreated and IPI-926 treated groups and all mice demonstrated engraftment of human leukemia by flow cytometry (Figure 5). To examine effects of short-term Hh inhibition on long-term self-renewal, we isolated human CD19^+^ cells from the bone marrow of mice from each treatment group and injected equivalent numbers of tumor cells into secondary recipients. Following 60 days, all mice receiving tumor cells from vehicle treated animals engrafted with leukemia. In contrast, leukemic engraftment was detected in only 1 of 5 recipient mice receiving bone marrow from IPI-926 treated donors (Table 2) although none of the secondary recipients received further treatment. These results demonstrate a persistent effect of Hh inhibition on the long-term self-renewing cells in B-ALL.

**Table 2:** Rates of secondary leukemia engraftment following *in vivo* treatment with IPI-926.

	Engraftment	Secondary engraftment
**IPI-926**	5/5	1/5
**Vehicle**	5/5	5/5

An initial cohort of mice was injected with REH cells and treated with either IPI-926 or vehicle control. All mice engrafted with leukemia. Following secondary transplantation of harvested bone marrow cells, 1 in 5 recipient mice of the bone marrow from IPI-926 treated mice engrafted with leukemia versus 5 in 5 recipient mice of vehicle-control (p<0.04).

### Hh signaling affects B-cell ALL self-renewal in a cell intrinsic manner

**Figure 6:**
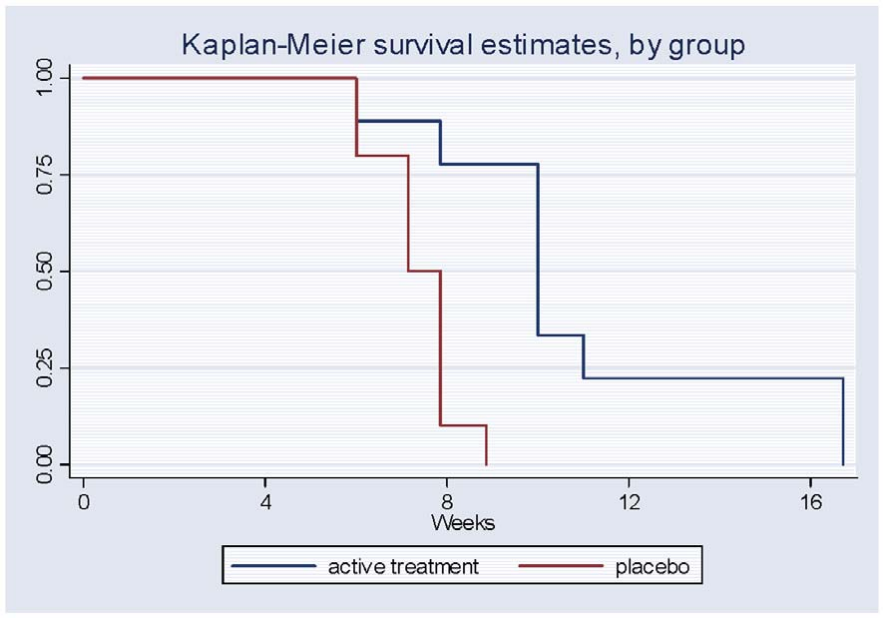
Prolonged overall survival of recipient mice of pre-treated IPI-926 REH cells. NOD-Scid mice were injected by tail vein with REH cells that had been pre-treated with either IPI-926 or vehicle *in vitro* prior to injection. Mice were followed for progression of symptoms and overall survival, and recipient mice of pre-treated cells had statistically significantly longer overall survival (70 days versus 50 days, p<0.001).

Recent data in mouse models of pancreatic and colon cancer have suggested that the Hh signaling pathway mediates interactions between tumor cells and the non-cancerous stromal cells in the microenvironment [29]. Here, tumor cells secrete Hh ligand that activates pathway signaling in neighboring stromal cells that in turn, are thought to elaborate factors promoting tumor cell growth and survival. Since our initial *in vivo* studies were unable to distinguish anti-tumor effects arising from pathway inhibition within tumor or stromal cells, we treated REH cells *in vitro* for two weeks with IPI-926, washed cells to remove drug, and then evaluated tumor growth in NOD/Scid mice. Although *in vitro* treatment did not inhibit B-ALL engraftment, mice injected with cells pre-treated with IPI-926 had a significantly prolonged overall survival (50 versus 70 days, p<0.001, Figure 6). Therefore, inhibition of the Hh pathway within B-ALL cells may directly impact tumor growth and disease progression.

## Discussion

Children and adults with newly diagnosed B-ALL respond equally well to induction chemotherapy with over 90% of patients achieving a complete remission. However, long-term outcomes markedly differ as the majority of children are cured whereas most adults relapse and die of progressive disease. Initial responses indicate that induction chemotherapy is effective at eliminating the majority of tumor cells, but subsequent relapse suggest that alternative means of inhibiting persistent leukemic cells capable of long-term self-renewal are needed.

In several human cancers, long-term growth potential appears to be restricted to distinct populations of CSC that share functional attributes with their normal counterparts, including self-renewal and the ability to give rise to differentiated progeny that form the tumor bulk. The identification of CSC in most human tumors has relied on distinct patterns of surface antigen expression. In B-ALL, the CSC surface phenotype is not clear since several unique markers have been used to isolate clonogenic cells.^16–21^ It is possible that specific antigen expression by CSC may vary among individual cases of B-ALL as a function of distinct genetic abnormalities [30]-[32] or stage of disease [33]. Rather than surface antigen expression, we examined intracellular ALDH activity since this approach can enrich for normal stem cells in several adult tissues [24], [25], [34]. Similar to several other malignancies, including MM, classical Hodgkin lymphoma, and breast and pancreatic carcinomas [6], [10], [27], [35], [36], we found that relative ALDH activity could enrich for B-ALL cells with increased growth potential. Moreover, ALDH^+^ cells displayed increased clonogenic expansion during serial passage compared to ALDH^neg^ cells suggesting that they had increased self-renewal potential.

Similar to normal stem cells, CSC self-renewal is required for long-term maintenance of the malignant clone. Therapeutic approaches that target self-renewal may improve long-term outcomes, but few approaches have been developed that target this property. Serial transplantation is widely accepted as an assay measuring long-term self-renewal in normal hematopoietic stem cells and other malignancies. We used a complementary *in vitro* assay with serial replating and colony-formation, as well as serial *in vivo* transplantation in order to assess the effects of Hh inhibition on long-term self-renewal. We found that the Hh pathway inhibitors 5E1, cyclopamine or the novel semi-synthetic cyclopamine derivative IPI-926 significantly reduced B-ALL cell self-renewal *in vitro* as well as during serial transplantation *in vivo*. This loss of serial transplantation ability is most consistent with self-renewal, as similarly seen in serial transplantation experiments with normal hematopoietic stem cells. It is notable that such long-lasting effects of Hh inhibition were seen following only short exposure to drug (either 72 hours in the colony-formation assays or 21 days of treatment in the primary recipient mice only). Based on our *in vitro* clonogenic data, we believe that this loss of serial colony-formation and transplantation ability is due to the effects of Hh inhibition on the self-renewal properties of B-ALL CSC. However, it is possible that the inhibition of engraftment during secondary transplantation is mediated by the effects of Hh inhibition on the quiescence of CSC, their ability to interact with potential stem cell niches, proper homing during transplantation, or the induction of terminal differentiation. Although cell reprogramming may be an interesting consideration, there is no current data to suggest that this that this process happens in any disease. Similar limitation of self-renewal following Hh inhibition has been reported for MM and CML [6], [18], [19], suggesting that the Hh signaling pathway may regulate self-renewing cells across several malignancies and represent a novel approach to therapeutically target long-term self-renewal.

Several models of Hh pathway activation in cancer have been described [37], [38]. Distinct tumors, such as BCC and medulloblastoma, may harbor mutations in *PTCH1* or *SMO* that leads to constitutive pathway activation in the absence of HH ligand [39], [40]. Autocrine signaling in which tumor cells both secrete and respond to ligand has also been described in a wide variety of human solid tumors [11], [14]–[16], [41]–[43]. In addition, recent data have suggested that paracrine Hh signaling may mediate interactions between tumor cells and non-transformed stromal cells through multiple mechanisms [5], [26], [41], [42]. In NHL and MM, stromal cells have been found to secrete HH ligand that promotes the survival of malignant plasma cells [8]. In pancreatic and colon carcinomas, tumor cells secrete ligand and stimulate Hh pathway activity in stromal cells to promote tumor growth [29], [44], [45]. This second paracrine model suggests that Hh signaling primarily occurs in normal, rather than tumor cells, but data in CML, colon cancer and our findings in B-ALL suggest that Hh signaling and its regulation of self-renewal is cell intrinsic [18], [19]. Both REH and RS4;11 cells were responsive to ligand stimulation as measured by increased *GLI1* expression as well as increased clonogenic growth. In these *in vitro* models, no stromal cells were used, and the effects of Hh activation by SHh and inhibition by 5E1, cyclopamine or IPI-926 were mediated through a cell intrinsic mechanism. Moreover, we found that *in vitro* treatment of REH cells with IPI-926 prior to injection into mice and in the absence of stromal cells significantly prolonged survival. The treatment of mice with IPI-926 following the injection of REH cells also had a profound effect on B-ALL cells, but primarily on their ability to undergo serial transplantation. Since B-ALL cells are heavily dependent on bone marrow stromal cells for their survival and growth, it is likely that the Hh signaling may occur through multiple modes in B-ALL.

In several human cancers, such as MM, the Hh signaling pathway may be active in only a portion of cases [6], [23]. In contrast, we found the expression of HH pathway components is a feature of both good risk (i.e., t(12;22) and poor risk (i.e., MLL-rearranged or Ph^+^) subtypes of B-ALL. Therefore, Hh signaling may represent a common therapeutic target in this disease, perhaps due to a role in early lymphoid development [7], [46]. Since our data suggest that the Hh pathway specifically targets the self-renewal of B-ALL cells, the optimal use of novel pathway inhibitors currently entering clinical testing may be as post-remission therapy following induction [47].

## Supporting Information

Table S1 **PCR primers.**
(DOCX)
