# Supplementary material for: Self-Renewal of Acute Lymphocytic Leukemia Cells Is Limited by the Hedgehog Pathway Inhibitors Cyclopamine and IPI-926
Source: PLoS One. 2010 Dec 28;5(12):e15262. doi: 10.1371/journal.pone.0015262 (PMC3011010; doi:10.1371/journal.pone.0015262)
Supplement: Table S1 — PCR primers. (DOCX) [file pone.0015262.s001.docx]

**Supplemental Table 1. PCR primers**

| Qualitative |  |  |  |
| --- | --- | --- | --- |
| Gene | Symbol | Genbank ID | Primer |
| *BETA ACTIN* | *ACTB* | NM_001101 | (+)ATCCACGAAACTACCTTCAACTCCATC |
|  |  |  | (-)CATACTCCTGCTTGCTGATCCACATC |
| *PATCHED1* | *PTCH* | NM_00264 | (+)AATAAGGCTGAGGTTGGTCATGGTTAC |
|  |  |  | (-)AGGGTCGTGGTGGTGAAGGAAAG |
| *PATCHED2* | *PTCH2* | NM_00378 | (+)ACTCTGGCTTCGTGCTTACTTCC |
|  |  |  | (-)TCCTGGCGTGCGGTCTGTATC |
| *SMOOTHENED* | *SMO* | NM_005631 | (+)GGTGTGGTTTGGTTTGTGGTCCTC |
|  |  |  | (-)CCTGGTTGAAGAAGTCGTAGAAGTGG |
| *GLI1* | *GLI1* | NM_005269 | (+)CCACGGGGAGCGGAAGGAG |
|  |  |  | (-)ACTGGCATTGCTGAAGGCTTTACTG |
| *GLI2* | *GLI2* | NM_005270 | (+)GAAGTTCGTGGACTCCTACAATAATGC |
|  |  |  | (-)GACTCACTGCTCTGCTTGTTCTGG |
| *GLI3* | *GLI3* | NM_005270 | (+)ATCCATCTCCGATTCCTCCATTGC |
|  |  |  | (-)GTATTCTGCTGGGCTGACTCCTG |
| *INDIAN* | *IHH* | 000074 | (+)GGCAGCTGTCTCTACACACG |
| *HEDGEHOG* |  |  | (-)GGGCCTAAGATGGATGGAAT |
| *SONIC* | *SHH* | NM_000193 | (+)CGGAGCGAGGAAGGGAAAG |
| *HEDGEHOG* |  |  | (-)TTGGGGATAAACTGCTTGTAGGC |
| *HEDGEHOG INTERACTING PROTEIN* | *HHIP* | NM_015690 | (+)CTCAAAGCCTGTTCCACTCACCTG |
|  |  |  | (-)TCCTCTTTCATCTCCTCCCTTTATTC |
|  |  |  |  |
|  |  |  |  |
| Quantitative |  |  |  |
| *BETA ACTIN* | *ACTB* | NM_001101 | (+)CATCCACGAAACTACCTTCAACTCC |
|  |  |  | (-)GAGCCGCCGATCCACACG |
| *PATCHED1* | *PTCH* | NM_00264 | (+)TTCCAGTTAATGACTCCCAAGCAAATG |
|  |  |  | (-)GCGACACTCTGATGAACCACCTC |
| *SMOOTHENED* | *SMO* | NM_005631 | (+)TGGTCACTCCCCTTTGTCCTCAC |
|  |  |  | (-)GCACGGTATCGGTAGTTCTTGTAGC |
| *GLI1* | *GLI1* | NM_005269 | (+)TTGGAGAAGCCGAGCCGAGTATC |
|  |  |  | (-)GAGTAGACAGAGGTTGGGAGGTAAGG |
|  |  |  |  |
|  |  |  |  |
